# Assessing the value of bypassing agent therapy used prophylactic versus on-demand, during immune tolerance induction for treatment of inhibitors: a retrospective chart review

**DOI:** 10.1186/s13023-023-02654-0

**Published:** 2023-03-07

**Authors:** George Morgan, Emily Back, Doug Rosa, Jamie O’Hara, Alan Finnegan

**Affiliations:** 1 HCD Economics, The Innovation Centre, Keckwick Lane, Daresbury, WA4 4FS UK; 2 Faculty of Health and Social Care, University of Chester, Chester, UK

**Keywords:** Haemophilia A, Factor VIII inhibitors, Factor VIII, Bypassing agents, Immune tolerance induction

## Abstract

**Background:**

Haemophilia A is a bleeding disorder caused by deficiency of coagulation factor VIII (FVIII) which leads to severe and repeated bleedings. There is a need to understand the optimal treatment pathway for FVIII inhibitors with the use of immune tolerance induction (ITI) and the role of haemostatic ‘bypassing’ agents (BPA) on-demand (OD) or prophylactically (Px). The aim of this study was to gain a better understanding of the real-world use of BPA therapy administered prophylactically or on-demand concomitant with ITI, for the treatment of an inhibitor to FVIII replacement therapy in patients with severe haemophilia A.

**Methods:**

Retrospective observational data were used to capture disease management information for patients who were aged 16 or under and had received ITI and BPA treatment for their most recent inhibitor from Jan-2015 to Jan-2019, for 47 patients in the UK and Germany. Descriptive comparisons of the clinical effectiveness and resource utilisation of Px and OD BPA therapy during ITI were conducted.

**Results:**

During ITI and BPA treatment, for an inhibitor, bleeding events averaged 1.5 and 1.2 for Px and OD treatment respectively. Compared to only BPA therapy we see 3.4 and 1.4 bleeding events for Px and OD respectively during an inhibitor.

**Conclusion:**

Baseline disease characteristics differed between BPA therapy cohorts and this resulted in higher clinical effectiveness of ITI treatment alongside BPA Px than BPA OD during an inhibitor.

## Introduction

For bleeding disorders, development of high-titre inhibitory antibodies are a significant complication as it limits the effectiveness of Factor VIII (FVIII) replacement therapy. Reducing the effectiveness of FVIII infusions can lead to clinical impacts such as muscle & joint complications plus higher mortality risk alongside monetary impact of increased healthcare costs [1–3]. The development of these alloantibody inhibitors are seen in 25–30% of patients against the infused Factor VIII, with the highest rates among children with severe haemophilia A [4]. To overcome an FVIII inhibitor they necessitate the chronic use of increasingly higher doses of FVIII in an attempt to achieve immune tolerance induction (ITI), or the use of haemostatic ‘bypassing’ agents (BPA) which is recommended either episodic or prophylactically with or without ITI [5].

The use of novel agents such as BPA on-demand (OD) or prophylactic (Px) during ITI has yet to be fully observed. Previous research has shown that BPA delivered prophylactically is more effective than on-demand in the reduction of bleeds per year, prevention of joint damage, reduction of hospital length of stay and improvement in quality of life [6, 7]. Non-factor treatment options such as Emicizumab and other novel therapies reaching the markets give hope that management of FVIII inhibitors won’t be needed for future generations. However, given a large proportion of people with haemophilia are still prescribed FVIII replacement therapy as their standard of care there is still a continued need to clarify and understand the optimal treatment pathway and the role of BPA, using evidence from real world practice.

The aim of this investigator-initiated retrospective chart review study is to contribute to the evidence generation for the use of BPA, OD or Px, concomitant with ITI. This study will quantify differences in outcomes of two separate patient populations with severe haemophilia A, already undergoing routine medical practice in their designed treatment centres, with one cohort receiving BPA Px alongside ITI for treatment of an inhibitor, and the second cohort receiving ITI only or with BPA OD. The real world data generated through the course of this non-interventional research study will fill a gap in the current evidence base and will contribute to efforts to optimise therapeutic approaches in severe haemophilia.

## Methods

### Data source and cohort collection

This investigator-initiated study was a retrospective, multicentre, observational chart review (case note review) of patients with severe haemophilia A who had a recorded incidence of an inhibitor to FVIII replacement therapy and were under 16 at that time, between the years 2015 and 2019. In addition to clinical and demographic information, data on treatment history prior to inhibitor diagnosis, and data on the use of ITI and BPA therapy for the treatment of the most recent inhibitor, were abstracted from patient medical records.

The study aimed to recruit 50 patients from two haemophilia treatment centres (HTCs), in the UK and Germany, Great Ormond Street Hospital and Universitaets Krankenhaus Bonn. The target sample size was determined by a feasibility assessment of the number of cases at participating HTCs within the defined time period. Data was generated from a retrospective review of patient medical records by clinical research nurses in partnership with the treating physicians. Investigators conducted an initial electronic screening of the records of patients treated by them at their institution in accordance with the study eligibility criteria. Investigators abstracted pseudonymised data from patient medical records into a standardized data collection tool. All abstracted data were checked by site investigators to ensure patient-identifiable information was removed ahead of the data being entered into the system. The pseudonymised data was then used to perform the statistical analyses to address the study objectives.

### Eligibility criteria

All patients included in the study had to have a diagnosis of severe haemophilia A which was confirmed prior to a diagnosis of an inhibitor to FVIII replacement therapy and must have had a confirmed history of an inhibitor to FVIII replacement therapy, in the period between January 2015 and January 2019. Participants had to be aged 16 or under at the time of their most recent inhibitor and received ITI treatment for their most recent inhibitor, alongside prophylactic BPA therapy or BPA therapy on-demand in the period between January 2015 and January 2019. If participants had previously received treatment with emicizumab, prior to or during the period of their most recent inhibitor they were declared to be not eligible for the study.

### Outcomes

The baseline characteristics collected on each participant included gender, country, ethnicity and age (years) at most recent inhibitor. In addition, the clinical outcomes collected at baseline included but were not exclusive to family history of inhibitors, number of bleeding events (bleeding events were defined as any type and of any severity) and joint bleeding prior to most recent inhibitor, number of target (3 + bleeds have occurred within a consecutive 6 month period) and problem joints (exhibiting symptoms of chronic damage due to haemophilia) prior to their most recent inhibitor, reason for development of most recent inhibitor and previous inhibitor diagnosis.

To analyse the clinical effectiveness of treatments for their most recent inhibitor; bleeding events and joint bleeding events during treatment for the inhibitor were analysed during BPA therapy and during ITI alongside BPA therapy. To analyse healthcare resource utilisation prior to inhibitor and during ITI alongside BPA therapy the number of hospital admissions and inpatient stay duration for bleed event reasons were analysed.

### Statistical methods for analysis

Variables derived from the study have been summarised using the following specifications.

- For continuous variables, means and standard deviations have been reported.
- For discrete variables, the frequency and proportions are reported and the number of observations per each variable have been reported.

This study did not impute any missing data. If data was missing at random for an individual participant, that observation was removed from the analysis.

Outcomes are presented according to treatment strategy; by the subgroups of BPA Px versus BPA OD for baseline characteristics prior to most recent inhibitor, by subgroups of ITI alongside BPA (Px and OD) versus BPA only (Px or OD) for clinical effectiveness and by subgroups of BPA (Px and OD) prior to inhibitor versus ITI alongside BPA (Px or OD) for resource utilisation. Where feasible, univariate comparisons between outcomes of interest have been tested for significance. Choice of significance test depended upon the nature of the variables tested, tests included Chi squared or fisher’s exact test for categorical variable comparisons (the latter for low frequency variables when one variable has n < 5) [8] and t-tests or non-parametric tests for comparing continuous variables (e.g. Wilcoxon signed rank sum test). All analyses were performed using STATA 17.0 statistical software (STAT Corp., College Station, TX) [9].

## Results

**Fig. 1:**
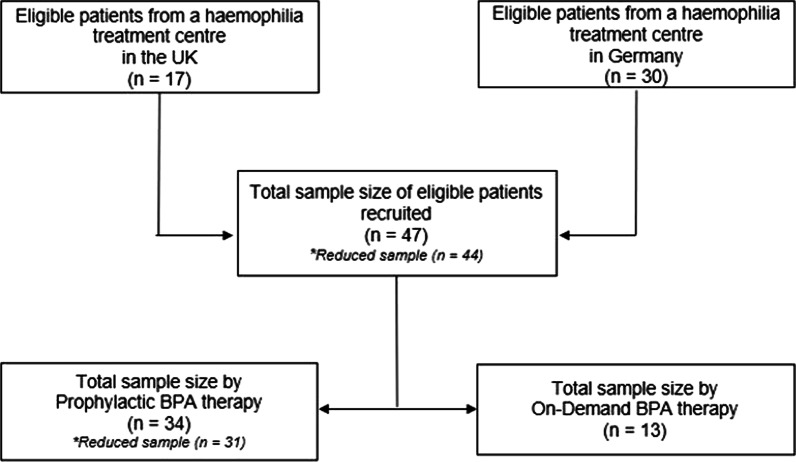
Flow Diagram of patient numbers. *n* (Sample size), *UK* (United Kingdom), *BPA* (Bypassing Agents). Total Sample size by prophylactic BPA therapy varies in the results due to missing data

The final sample consisted of 47 male patients with severe haemophilia A, 28% (n = 13) receiving on-demand treatment and 72% (n = 34) receiving prophylaxis treatment. A flow diagram of patient numbers is presented in Fig. 1.

### Baseline characteristics

The baseline characteristics of the patients are presented in Table 1 by the BPA therapy subgroups. Age at time of most recent inhibitor averaged of 10.6 years (SD: 4.5) with similar subgroup mean ages for both BPA Px (10.6 years, SD: 4.7) and BPA OD (10.5 years, SD: 4.1). Most participants were of white ethnicity with 90% for the BPA Px group and 92% for the BPA OD sample. A difference was seen between the samples in reporting a family history of an inhibitor with the BPA Px sample reporting 45% and BPA OD reporting 62%, however this was not statistically significant.

**Table 1:** Summary baseline characteristics of participants with severe haemophilia A by treatment regimen

Baseline characteristics	Sample size as above unless stated otherwise in the table			
	BPA treatment type		Total	Statistical significance
	Prophylactic (*n* = 34)	On-demand (*n* = 13)	*n* = 47	p-value* (< 0.05)
Gender *n* (%)				
Male	34 (100)	13 (100)	47 (100)	
Country, *n* (%)				
Germany	20 (59)	10 (77)	30 (64)	
UK	10 (41)	3 (23)	17 (36)	
Ethnicity, *n* (%)				
White	30 (90)	12 (92)	42 (90)	
Black	3 (9)	0 (0)	3 (6)	
Mixed	1 (1)	1 (8)	2 (4)	
Age most recent inhibitor (years), mean ± SD	10.6 ± 4.7	10.5 ± 4.1	10.6 ± 4.5	0.952
Family history of an inhibitor, *n* (%)	*n* = 33	*n* = 13	*n* = 46	
Yes	15 (45)	8 (62)	23 (50)	0.326
No	18 (55)	5 (38)	23 (50)	
Bleeding events in last 12 months prior to most recent inhibitor, mean ± SD	*n* = 31 1.9 ± 1.4	*n* = 13 1.4 ± 1.1	*n* = 44 1.7 ± 1.2	0.349
Joint bleeding events in last 12 months prior to most recent inhibitor, mean ± SD	*n* = 31 1.4 ± 1.4	*n* = 13 1.2 ± 1.0	*n* = 44 1.3 ± 1.2	0.914
Target joints, *n* (%)				
Yes	31 (91)	13 (100)	44 (94)	
No	3 (9)	0 (0)	3 (6)	0.369
Problem joints, *n* (%)				
Yes	31 (91)	13 (100)	44 (94)	
No	3 (9)	0 (0)	3 (6)	0.369
Frequency of target joints, mean ± SD	6.8 ± 4.3	9.1 ± 3.4	7.4 ± 4.1	0.059
Frequency of problem joints, mean ± SD	6.6 ± 4.3	8.6 ± 3.5	7.2 ± 4.1	0.131

*n* Sample size; *mean* Average value; *SD* Standard deviation; *ITI* Immune tolerance induction; *BPA* Bypassing agent

*p-value < 0.05 (statistically significant)

**Fig. 2:**
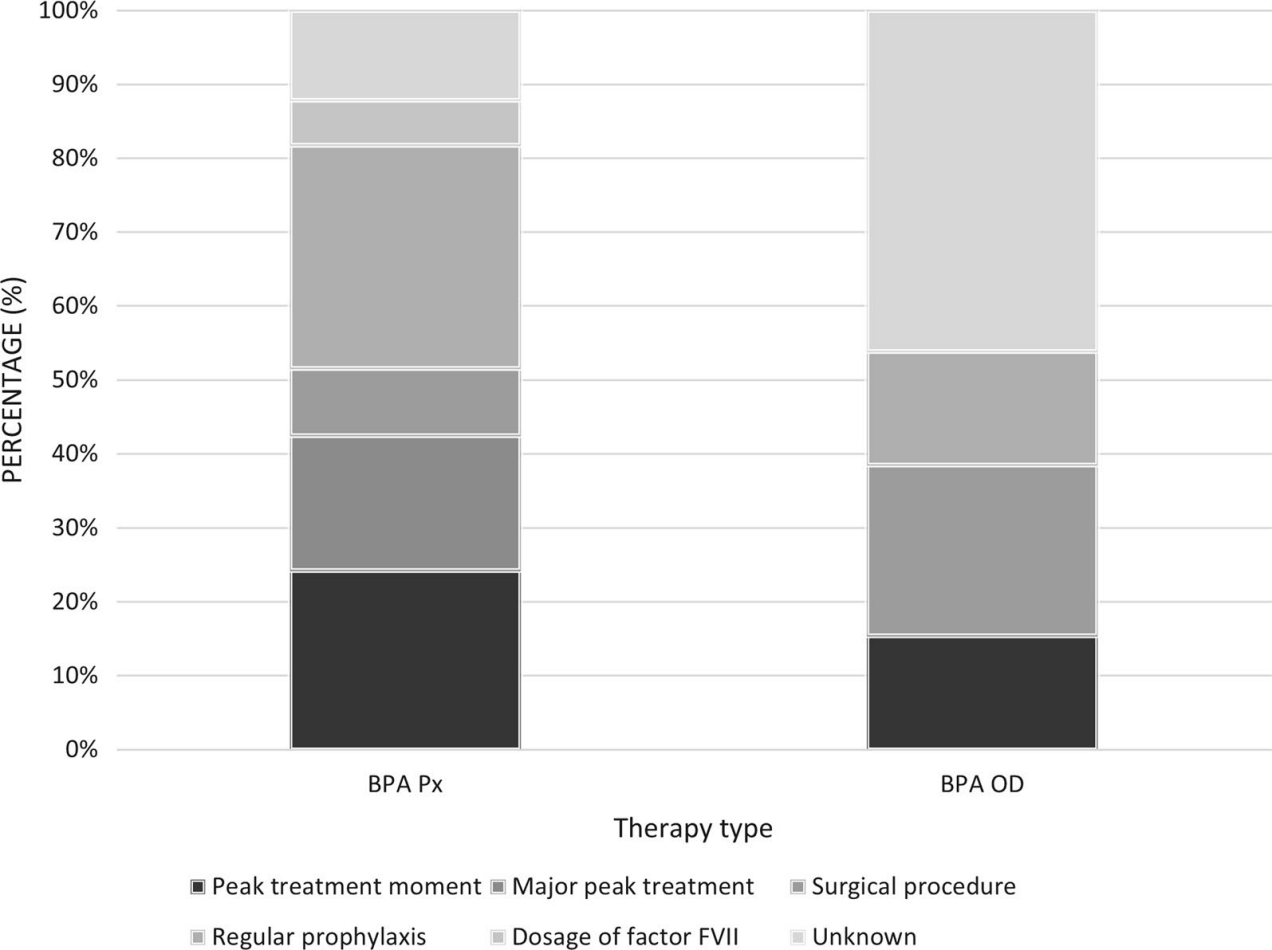
Reason for development of most recent inhibitor by BPA therapy. *BPA* (Bypass Agent), *BPA Px* (Bypass Agent Prophylaxis), *BPA OD* (Bypass Agent On-Demand)

The most common reason for an inhibitor developing was related to regular prophylaxis for the BPA Px cohort (*n* = 10, 30%) and for the BPA OD cohort the most common reason for an inhibitor developing remained unknown (*n* = 6, 47%), followed by surgical procedure (*n* = 3, 23%) as presented in Fig. 2. Overall, most patients reported being diagnosed once (*n* = 33, 72%) indicating this was their first inhibitor, whereas 28% respondents reported being diagnosed more than once in their lifetime (*n* = 13, 28%) therefore having a separate inhibitor prior to their most recent. For the BPA Px subgroup 76% (n = 25) of the prophylaxis were diagnosed with inhibitor once and for the on-demand, 62% (n = 8) were diagnosed with inhibitor once in their lifetime.

On average, prior to most recent inhibitor, there were 1.9 bleeding events in last 12 months for the BPA Px cohort (*n* = 31, SD: 1.4) compared to 1.4 bleeding events in last 12 months for BPA OD cohort (n = 13, SD: 1.1). Similarly, the BPA Px group had on average 1.4 joint bleeding events in last 12 months (*n* = 31, SD: 1.4) prior to most recent inhibitor whereas BPA OD had an average of 1.2 (n = 13, SD: 1.2). Overall, 94% of the respondents reported having target joints and problem joints, with 91% (n = 31) and 100% (n = 13) respectively for the BPA Px and BPA OD cohorts. Overall, the mean frequency of target joint locations for respondents was 7.4 (n = 47, SD: 4.1), with a mean of 6.8 (n = 34, SD: 4.3) for the BPA Px sample and 9.1 (n = 13, SD: 3.4) for the BPA OD sample. The mean frequency of problem joints was lower in comparison to target joints, with 7.2 (SD: 4.1) overall, and 6.6 (SD: 4.3) for BPA Px and 8.6 (SD: 3.5) for BPA OD.

### Clinical effectiveness outcomes

The clinical effectiveness of ITI alongside BPA (Px or OD) and BPA (Px or OD) only for treatment of the most recent inhibitor are presented in Table 2. On average, during ITI treatment, bleeding events of any type and of any severity occurred 1.4 times (*n* = 44, SD: 1.3), with an average of 1.5 (n = 31, SD: 1.5) and 1.2 (n = 13, SD: 1.2) for ITI BPA Px and ITI BPA OD respectively. Compared to only BPA therapy we see there was an average of 2.8 bleeding events reported overall, with 3.4 bleeding events for the BPA Px (*n* = 31, SD: 5.5) and 1.4 events (*n* = 13, SD: 0.8) for the BPA OD during the inhibitor. Joint bleeding events occurred 1.2 times overall (*n* = 44, SD: 1.6), with an average of 1.3 (n = 31, SD: 1.8) and 1.1 (n = 13, SD: 0.8) during ITI BPA Px and ITI BPA OD respectively. In comparison to BPA only, joint bleeding events of any severity averaged 2.1 times overall (*n* = 44, SD: 3.6) with 2.5 (n = 31, SD: 4.2) and 1.2 (n = 13, SD: 0.8) for BPA Px and BPA OD respectively.

**Table 2:** Clinical effectiveness of BPA during ITI therapy of patients with severe haemophilia A by treatment type

Clinical effectiveness	Outcomes during ITI alongside BPA				Outcomes during BPA only			
	BPA treatment type		Total	Statistical significance	BPA treatment type		Total	Statistical significance
	Prophylactic (*n* = 31)	On-demand (*n* = 13)	*n* = 44	*p*-value* (< 0.05)	Prophylactic (*n* = 31)	On-demand (*n* = 13)	*n* = 44	*p*-value* (< 0.05)
Bleeding events (any type and of any severity), mean ± SD	1.5 ± 1.5	1.2 ± 0.8	1.4 ± 1.3	0.830	3.4 ± 5.5	1.4 ± 0.8	2.8 ± 4.7	0.191
Joint bleeding events (of any severity), mean ± SD	1.3 ± 1.8	1.1 ± 0.8	1.2 ± 1.6	0.746	2.5 ± 4.2	1.2 ± 0.8	2.1 ± 3.6	0.631

*n* Sample size; *mean* Average value; *SD* Standard deviation; *ITI* Immune tolerance induction; *BPA* Bypassing agent

*p-value < 0.05 (statistically significant)

### Hospital resource utilisation

**Fig. 3:**
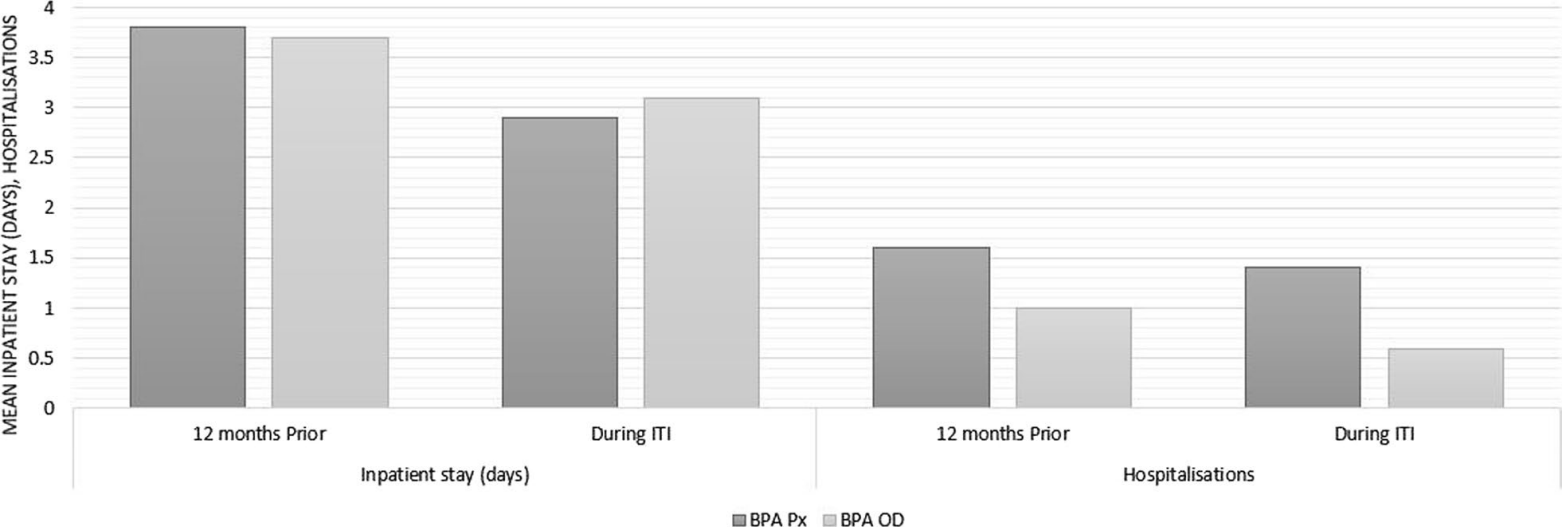
Resource utilisation in terms of inpatient stays and hospitalisations prior to inhibitor and during ITI by BPA therapy cohorts. *ITI* ((Immune Tolerance Induction), *BPA* (Bypass Agent), *BPA Px* (Bypass Agent Prophylaxis), *BPA OD* (Bypass Agent On-Demand)

Healthcare resource utilisation prior to inhibitor compared to during ITI alongside BPA therapy is presented in Fig. 3. Overall, in the 12 months prior to the most recent inhibitor, the number of hospitalisations relating to haemophilia was 1.4 times (n = 44, SD: 1.2). Patients who received BPA Px during an inhibitor were hospitalised 1.6 times (n = 31, SD: 1.3). Whereas those who received BPA OD during an inhibitor were hospitalised 1.0 time (n = 13, SD: 1.1). Inpatient length of stay was consistent between the two cohorts with an average of around 3.8 days (n = 44, SD: 4.0). During an inhibitor and receiving ITI alongside BPA therapy, there was on average 1.25 (n = 44, SD: 1.5) hospitalisations, with an average of 1.48 (n = 31, SD: 1.7) for the ITI BPA Px cohort and an average of 0.69 (n = 13, SD: 0.7) for the ITI BPA OD cohort. Inpatient stay in days was consistent between the two cohorts and overall, with an average of around 3.0 days (n = 44, SD: 3.5).

## Discussion

This investigator-initiated retrospective chart review study aimed to examine the efficacy of BPA therapy administered prophylactically or on-demand, in a real-world context, concomitant with ITI treatment for an inhibitor to FVIII replacement therapy in patients with severe haemophilia A.

This study compared key baseline characteristics of the BPA therapy subgroups of Px and OD for the treatment of BPA during ITI and BPA only. The baseline demographic characteristics were overall similar but differences were seen in the disease characteristics across groups. Specifically, a difference was seen between the samples in reporting a family history with the BPA Px cohort (45%) having a much lower percentage compared to the BPA OD cohort (62%). In addition, the reason for inhibitor development varied and bleeding events prior to their most recent inhibitor were consistently higher on average for the BPA Px cohort than for BPA OD (1.9 vs 1.4 bleeds). The study highlighted that prior and during an inhibitor event, bleeding episodes were also higher during BPA Px therapy compared to BPA OD regardless of BPA therapy alongside ITI (1.5 vs 1.2 bleeds) or BPA treatment only (3.4 vs 1.4 bleeds). However, when comparing the BPA Px cohort with and without ITI during an inhibitor the results show a significant reduction in bleed events using ITI alongside BPA Px (1.5 vs 3.4 bleeds). Related-hospitalisations rates were also higher for BPA Px than BPA OD at baseline (1.6 vs 1.0 visits) and during their most recent inhibitor (1.4 and 0.6 visits).

Although the results of this study show that the prophylaxis cohort had the highest bleed rates, other studies in the literature indicate different outcomes. One study reported the bleeding rates of prophylaxis and on-demand cohorts with BPA for six months, and found that with the prophylaxis BPA cohort bleeding rates were significantly reduced compared to the on-demand cohort [10]. Similarly, according to the literature, patients with Haemophilia A and inhibitors to FVIII who experience acute bleeding events during ITI and are managed with on-demand bypassing therapies will almost inevitably lead to the development and/or worsening of haemophilic arthropathy [11]. Looking at the baseline results of the patients in this study, the prophylaxis cohort had higher mean bleeding and joint bleeding events prior to the most recent inhibitor (as shown in Table 1). This could partially explain the higher bleeding averages for the prophylaxis cohort in comparison to the on-demand treatment group.

There is limited literature available that specifically evaluates the use of prophylaxis with BPA prior to or while patients are receiving ITI and to the authors’ best knowledge, no comparative trials of bypassing agents used as prophylaxis in patients with Haemophilia A and inhibitors to FVIII are currently available in the literature. Evidence from the study data that is available has demonstrated that concomitant administration of BPA prophylaxis during ITI leads to a decrease in bleeding events [11–13]. Published case reports second this by providing evidence of successful use of prophylaxis in patients prior to or during ITI, contradictory to our findings. [11, 14–22].

It is important to understand the results of this study within the context of existing evidence. It is likely that the treatment pathway of BPA concomitant with ITI is provided to patients of a greater severity, therefore it could be inferred that this study’s results show more severe outcomes for BPA in comparison to ITI as this higher level of severity may act as a confounder for the analysis. This is mirrored in the outcomes between the prophylaxis and on-demand cohorts. Additionally, patients have been found to have less predictable responses to bypassing agents compared with factor replacement therapy and some inhibitor patients respond better to some bypass agents compared to others during serious bleed events [11, 23]. So, although it can be inferred that this study shows that the treatment pathway of BPA concomitant with ITI results in a higher bleeding and joint bleeding rate on average during treatment for an inhibitor to FVIII replacement therapy, there are multiple contextual factors to take into consideration that may support the opposite claim.

### Strengths and limitations

There is a continued need to clarify and understand the optimal treatment pathway for patients with Haemophilia A, and the role of BPA using evidence from real world practice. This study helps to understand the clinical value of BPA prophylaxis during ITI and therefore contributes evidence for an area of limited research. The real-world data generated through the course of this non-interventional research study will help fill a gap in the current evidence base and will contribute to efforts to optimise therapeutic approaches in severe haemophilia.

There are inherent limitations related to the retrospective observational chart reviews of this study; this methodology could only provide a retrospective insight into the treatment pathways by reviewing. Although ITI has been demonstrated to have a response rate of 50–75%, it requires long term infusion and can take years to show its effect on clinical outcomes [24]. Future research in the form of a longitudinal study may capture the full patient story and the long-term efficacy of BPA concomitant with ITI treatment better. As this was a descriptive comparison, controlling for confounding variables and use of more sophisticated statistical methods should be considered in future work. Furthermore, due to Haemophilia being a rare condition, this study was limited by sample size in our subgroup analysis. There was a large difference in sample size between subgroups (n = 31 vs n = 13) as the prophylaxis group had more than double the sample size of the on-demand group. The small sample has likely led to most outcomes being not statistically significant.

## Conclusion

Our findings indicated that patients with BPA therapy administered prophylactically or on-demand, concomitant with ITI treatment for an inhibitor to FVIII replacement therapy reduced bleeding events compared to BPA treatment only. A future longitudinal study would best capture the long-term efficacy of BPA concomitant with ITI treatment. Although Haemophilia A is a rare condition, if possible, a larger more equitable sample size between the prophylaxis and on-demand cohorts spanning a larger recruitment window may provide more statistically significant results and an accurate depiction of the efficacy of the treatment pathways.

## Data Availability

All data relevant to the study are included in the article or uploaded as supplementary information.

## Abbreviations

BPA: Bypassing agent
FVIII: Factor VIII
HTC: Haemophilia treatment centre
ITI: Immune tolerance induction
Mean: Average value
N: Sample size
OD: On demand
Px: Prophylactic
SD: Standard deviation
